# The skiers knee without swelling or instability, a difficult diagnosis: a case report

**DOI:** 10.1186/1752-1947-1-11

**Published:** 2007-04-20

**Authors:** Mark E O'Donnell, Stephen A Badger, David Campbell, Willie Loan, Brendan Sinnott

**Affiliations:** 1 Department of Surgery, Belfast City Hospital, Lisburn Road, Belfast BT9 7AB. Northern Ireland, UK; 2 Department of Radiology, Belfast City Hospital, Lisburn Road, Belfast BT9 7AB. Northern Ireland, UK; 3 Department of Emergency Medicine, Belfast City Hospital, Lisburn Road, Belfast BT9 7AB, Northern Ireland, UK; 4 DSEM MFSEM(UK) MRCSEd, 42 Woodrow Gardens, Saintfield, Co Down, BT24 7WG, Northern Ireland, UK

## Abstract

Skiing as a recreational activity has increased exponentially in the last twenty-years. Similar to any sporting activity, participants can sustain various types of injury, which provides the emergency departments with a continuous supply of patients. The injury pattern from the slopes has also changed over this time period, due to alterations and improvements in ski equipment. An increased diversity in alpine skiing techniques, as well as snowboarding and cross-terrain disciplines has also influenced this change.

We present a multi-media experience of a high-speed ski fall that caused a valgus-external rotation injury to the right knee that precluded the patient from further ski activity. There was no bruising, swelling or instability demonstrated and the patient returned to ski activities 24-hours post-injury. Although this injury appeared clinically benign initially, the patient complained of persistent pain around the right knee which was causing occupational difficulties. Following normal clinical assessment, the patient returned to work but continued to complain of persistent pain at the lateral aspect of the right knee. Magnetic Resonance Imaging (MRI) demonstrated extensive bone marrow oedema (BMO), a mild depression of the articular cortex compression with a small focus of articular cartilage disruption and microfractures of the lateral tibial plateau. The patient was treated conservatively and remains well with avoidance of impact exercises 14-months post-injury.

In the presence of any high speed injury, we would stress that regardless of initial normal investigations, clinical suspicion should remain paramount and not deter the physician from further investigation in the presence of continuing symptomatology.

## Background

A 30 year-old male presented to the Accident and Emergency Department in March 2006 with persisting right knee pain. He described a high-speed fall during a ski holiday in January 2006 in which he sustained a valgus-external rotation knee injury (Additional file 1). His height was 168 cm and weight was 74 kg. He had a background of Alpine Ski Racing and stated that this was "the worst fall in 27-years and that he was unable to continue skiing". On assessment at the resort, maximal tenderness was elicited around the lateral tibial plateau and mid-tibial region. However, there was no evidence of bruising, swelling or joint instability. He was otherwise well with no previous history of trauma or musculoskeletal injuries. He was commenced on regular non-steroidal analgesics and returned to the slopes after a 24-hour rest period with a semi-rigid protection knee brace.

**Figure 1:**
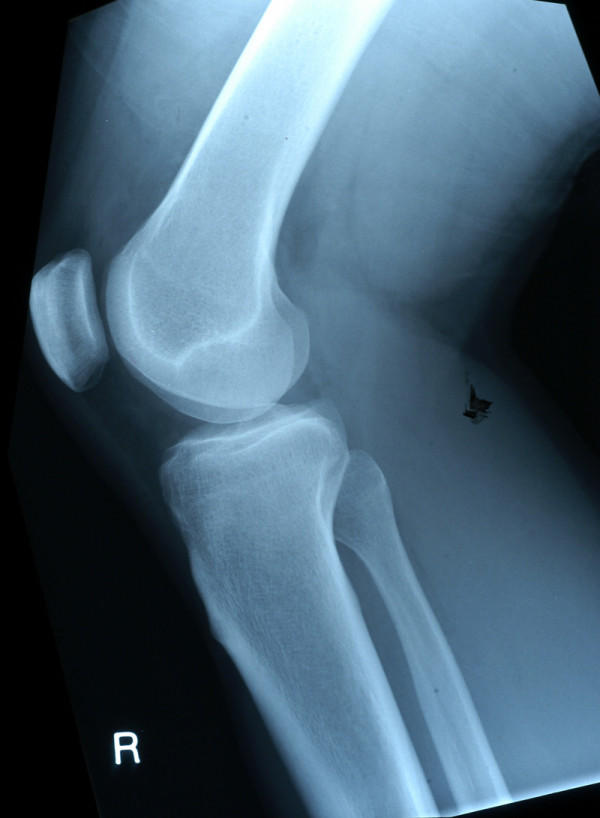
Plain Lateral Radiograph of the right knee demonstrating no obvious bony injury.

**Figure 2:**
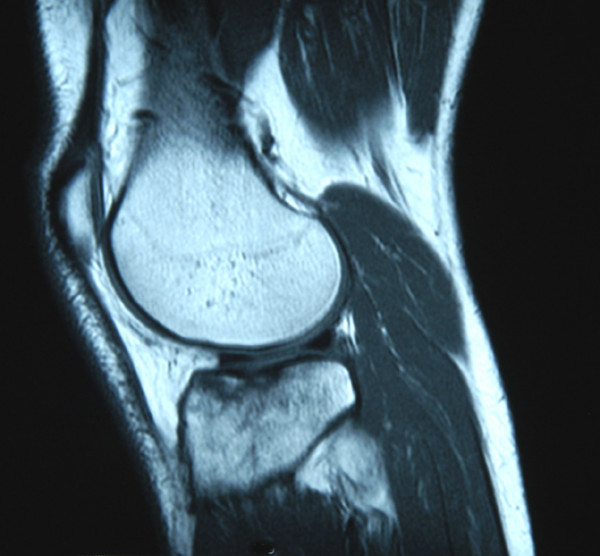
Sagittal T1-weighted sequence MRI demonstrating extensive bone marrow oedema within the lateral tibial plateau extending to the articular cartilage.

**Figure 3:**
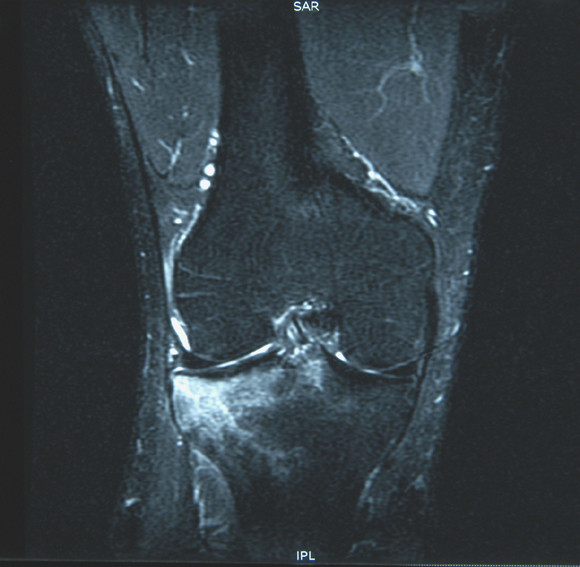
Coronal STIR sequence MRI demonstrating extensive bone marrow oedema within the lateral tibial plateau extending to the articular cartilage.

On return from the holiday, the patient described persistent pain around the lateral aspect of the knee joint radiating down to the mid-tibial region which was exacerbated by prolonged standing. The discomfort from the injury was now precluding him from his occupation which involved prolonged procedures in the standing position. On assessment in the Accident and Emergency Department 6-weeks following the injury, tenderness was again elicited around the lateral aspect of the knee and mid-tibia with no clinical evidence of a haemarthrosis or joint instability. Plain radiographs of the knee joint were normal (Figure 1 – Lateral View). The pain continued to persist necessitating further investigation. MRI demonstrated extensive BMO within the lateral tibial plateau extending to the articular surface and involving the tibial spine. There was a mild depression of the articular cortex of the lateral tibial plateau anteriorly with further compression microfractures in the deeper trabecular bone. There was also a small focus of articular cartilage disruption but no major cartilaginous defect (Figures 2 &3).

Immobilization of the joint in the post-injury period was advised by the othopaedic team. As it was more than 6-weeks following the injury, he was therefore advised to mobilise as tolerated with an arthropad support. The patient remains well 14-months following the injury and has minimal difficulty on prolonged standing. Impact training still exacerbates the discomfort around the right knee joint, and he prefers to continue with non-impact exercises.

## Discussion

There has been an increase in total ski injuries treated within emergency units over the last 20-years reflecting the unprecedented increase in alpine skiing popularity. In 2002, an estimated 77,300 skiing- and 62,000 snowboarding-related injuries were treated in the US hospital emergency departments [1]. However, with improved safety of ski equipment for everyone from the novice beginner to the more adept racer, the actual incidence has declined in this period. Although there has been a 60% decrease in total lower extremity injury incidence, knee ligamentous injuries still occur frequently and account for the largest percentage of lower limb injuries [2]. Most injuries occur in male participants between the ages of 10–24 years of age. However, Xiang *et al *state that the actual higher rate occurs among skiers aged 55–64 when injury analysis is completed correlating for the actual number of participants for each age group [1].

The clinical history of knee injury in skiers will often present the diagnosis with 90% accuracy [2]. Various factors guide the clinician to the diagnosis such as the force and type of fall (twisting, hyperextension, or falling backward), whether a "pop" was heard by the skier, the presence of instability after the fall and any associated swelling. In this case, the patient complained of pain following an external-rotation injury and described no actual feeling of joint instability or swelling. The mechanism of most knee injuries, as in this case, usually involves fixation of the distal extremity resulting in subsequent enhancement of the forces necessary to generate an injury.

The conventional radiographic knee series has traditionally been the method of choice in the initial radiological evaluation of patients with acute knee injury. The images required in a knee series varies between institutions from 2 to 6 images with no conclusive algorithm depicting which views should be included. In our institution, only antero-posterior (AP) and lateral knee views are recorded. Verma *et al *describe a lateral view as the most appropriate radiograph for screening knee trauma with a sensitivity for depicting knee fractures of 100%, and a negative predictive value of 100% in their study population with a 24.8% prevalence of knee fractures [3]. Although specificity was not the aim of Verma's study, they recommended MR imaging if clinical suspicion persisted in the present of inequitable radiograms. In this case, both AP and lateral views demonstrated no obvious bony injury. However, with the continuation of symptoms MR imaging was subsequently performed which demonstrated extensive BMO, a depression fracture of the articular cortex and further compression microfractures within the lateral tibial plateau. BMO secondary to sporting activities is most frequently related to trauma with an incidence between 27% and 72% after acute injuries [4]. The pathophysiology of BMO relates to the actual mechanism of injury and can occur in the acute or chronic setting. Impaction injuries, as seen in this case, are due to direct trauma which causes one bone to impact on another and results in extensive BMO involving a broad surface of the involved bony structures. Avulsive or distraction injuries are usually due to valgus, varus rotational stresses on a joint which causes a small avulsion fracture related to a tendinous, ligamentous or capsular attachment on the bone. The resultant BMO is less extensive as cortical rather than trabecular bone is involved. It is important to note that conventional X-rays are often more beneficial in these cases as the avulsed fragment may be very difficult to detect on MRI. Clinically, a combination of BMO patterns is usually evident as the impaction type is encountered at the force entry site whereas the distraction type is identified with possible underlying ligamentous injury at the force exit site [4].

Injuries that normally require surgical intervention include meniscal tears, anterior cruciate ligament tears, chrondral defects, and less often collateral ligament injuries. [1] The management of tibial plateau fractures on the other hand is a long subject of controversy. The spectrum of treatment ranges from simple casting and bracing to skeletal traction and early motion to open reduction and internal fixation (ORIF) [5]. Ebraheim *et al's *indication for surgery was dependant on the patient's age, medical status, presence of osteoporesis, degree of displacement and depression, pre-injury activity level, and occupation rather than solely on the Schatzker classification [6] for tibial plateau fractures. As the largest operative series to date, Ebraheim *et al *recommend ORIF only for those tibial plateau fractures with significant displacement [5].

The extensive BMO with associated microfractures identified on the MRI are synonymously known as bone bruises or contusions which are often used as secondary signs for detecting other associated abnormalities. They are best diagnosed by MRI with an increased signal intensity on fat suppressed T2-weighted images (eg. Short Tau Inversion Recovery, or STIR), and decreased signal intensity on T1-weighted images. T2-weighted images reflect the presence of free water (oedema or inflammatory response) and haemorrhage and are therefore useful to determine how acute the injury is. Bone bruises can be classified using MRI as Type I when the injury is diffuse, often reticular with alterations to the medullary component distant from the subadjacent articular surface, Type II which is defined as localized or geographical signal abnormality with contiguity to articular surface and type III as disruption or depression of the normal contour of the cortical surface/subchondral lamella, often associated with a Type II lesion [7].

In this case, MRI demonstrated minimal displacement of the articular cortex and ORIF was not indicated. The clinical significance, time required for resolution, long-term consequences to articular cartilage and the most appropriate initial treatment for bone bruises still await long-term follow-up studies. However, shorter-term studies reviewed by Mandalia *et al *stated that bone bruising has a variable time for resolution from as early as 3-weeks to 2-years [8]. They also suggested that bone bruising and other associated injuries can lead to deleterious effects on future cartilaginous metabolism and osteoarthritis in the longer term. However, these factors still require further study.

## Conclusion

The presence of persistence knee pain following a high-speed injury should alert the physician to consider further investigations even in the absence of obvious clinical signs or radiological findings. Treatment should be symptomatic with an initial period of immobilization recommended.

## Competing interests

The author(s) declare that they have no competing interests.

## Authors' contributions

**MEOD**: Involved in the conception of the report, literature review, manuscript preparation, manuscript editing and manuscript submission. **SAB**: Involved in the manuscript preparation and manuscript editing. **DC**: Involved in the critical analysis of radiological imaging in both the case report and discussion, manuscript editing and manuscript review. **WL**: Involved in the manuscript editing and manuscript review. **BL**: Involved in the critical review of the literature for sporting injuries of the knee, manuscript editing and manuscript review. All authors have read and approved the final manuscript.

## Supplementary Material

Additional File 1 Reduced speed footage documenting the actual valgus-external rotation force on the right knee. Click here for file
